# Supplementary material for: Dynamic allelic expression in mouse mammary glands across the adult developmental cycle
Source: Nucleic Acids Res. 2025 Sep 9;53(17):gkaf804. doi: 10.1093/nar/gkaf804 (PMC12419806; doi:10.1093/nar/gkaf804)
Supplement: gkaf804_Supplemental_Files [file gkaf804_supplemental_files.zip › Supplementary figures - Dynamic allelic expression in mouse mammary gland_revised.pdf]

Dynamic allelic expression in mouse mammary gland across the adult developmental cycle

Supplementary data

Supplementary Figure 1 – Representative wholemount images of mammary glands at selected developmental stages.

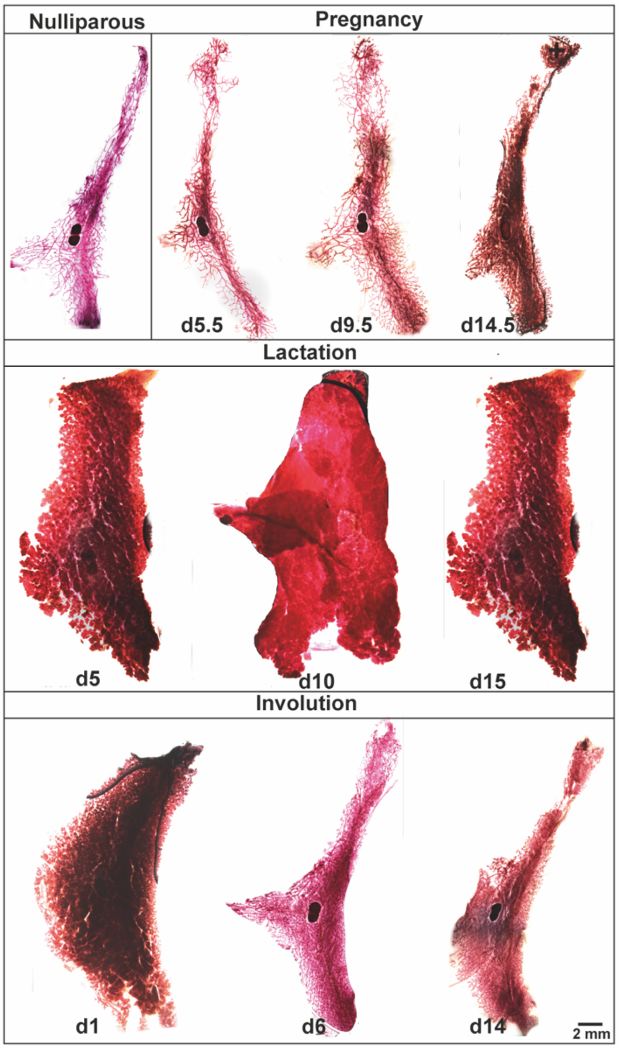

Carmine alum stained wholemounts of dissected mammary glands at the same time points showing the typical development of the tissue, showing entire images of abdominal mammary glands.

Supplementary Figure 2 – Gating strategy and representative flow cytometry plots.

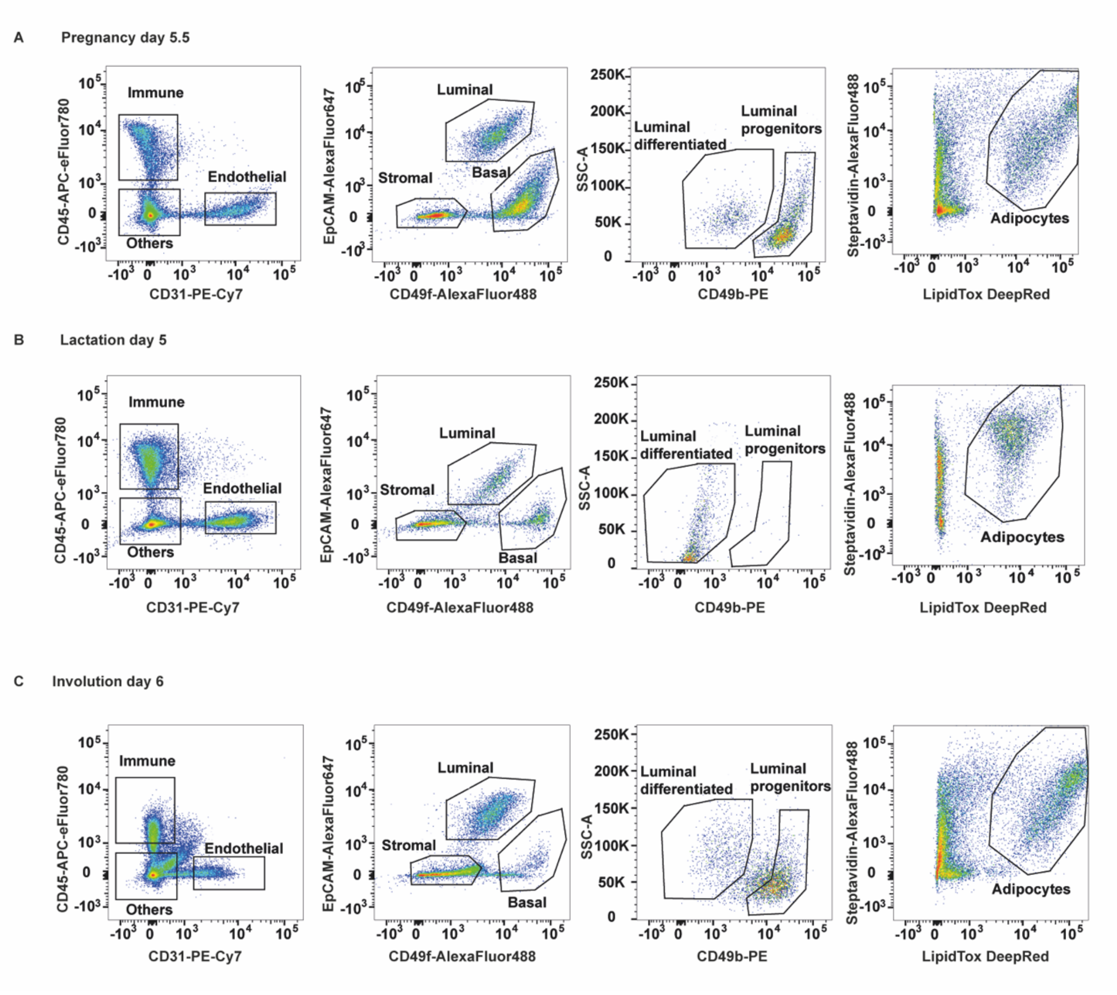

A-C Representative flow cytometry dot plot for pregnancy day 5.5 **(A)**, lactation day 5 **(B)**, and involution day 6 **(C)** mammary glands showing the isolated endothelial, luminal, basal, stromal, and adipocyte cell populations.

Supplementary Figure 3 – RNA quality assessment for RNA used in transcriptomic workflows.

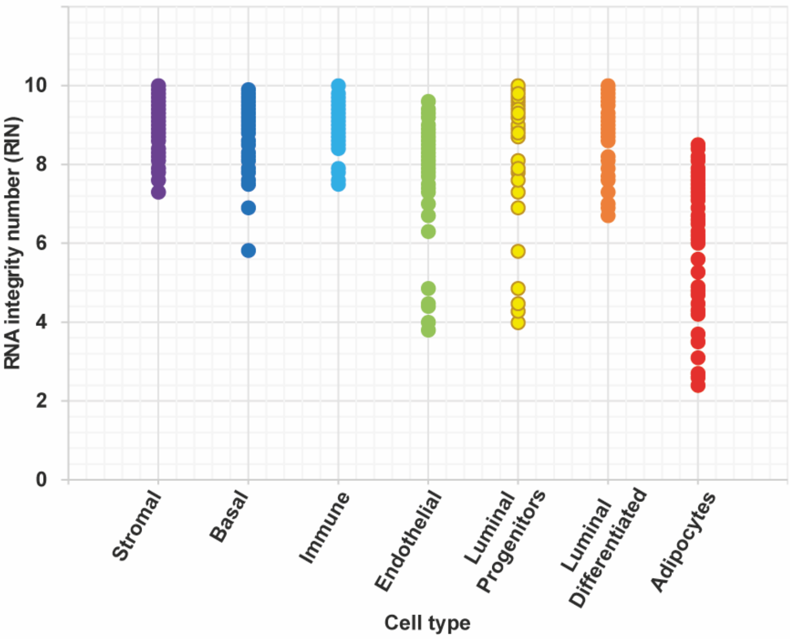

RNA integrity number of 80 sorted cell populations used in this study, including stromal, basal, immune, endothelial, luminal progenitor and differentiated cells and mammary adipocytes. Each dot represents the RNA integrity number of a population used for RNA-seq library preparation.

Supplementary Figure 4 – Allelic expression bias of imprinted genes by genomic position and developmental stage in mammary cell types.

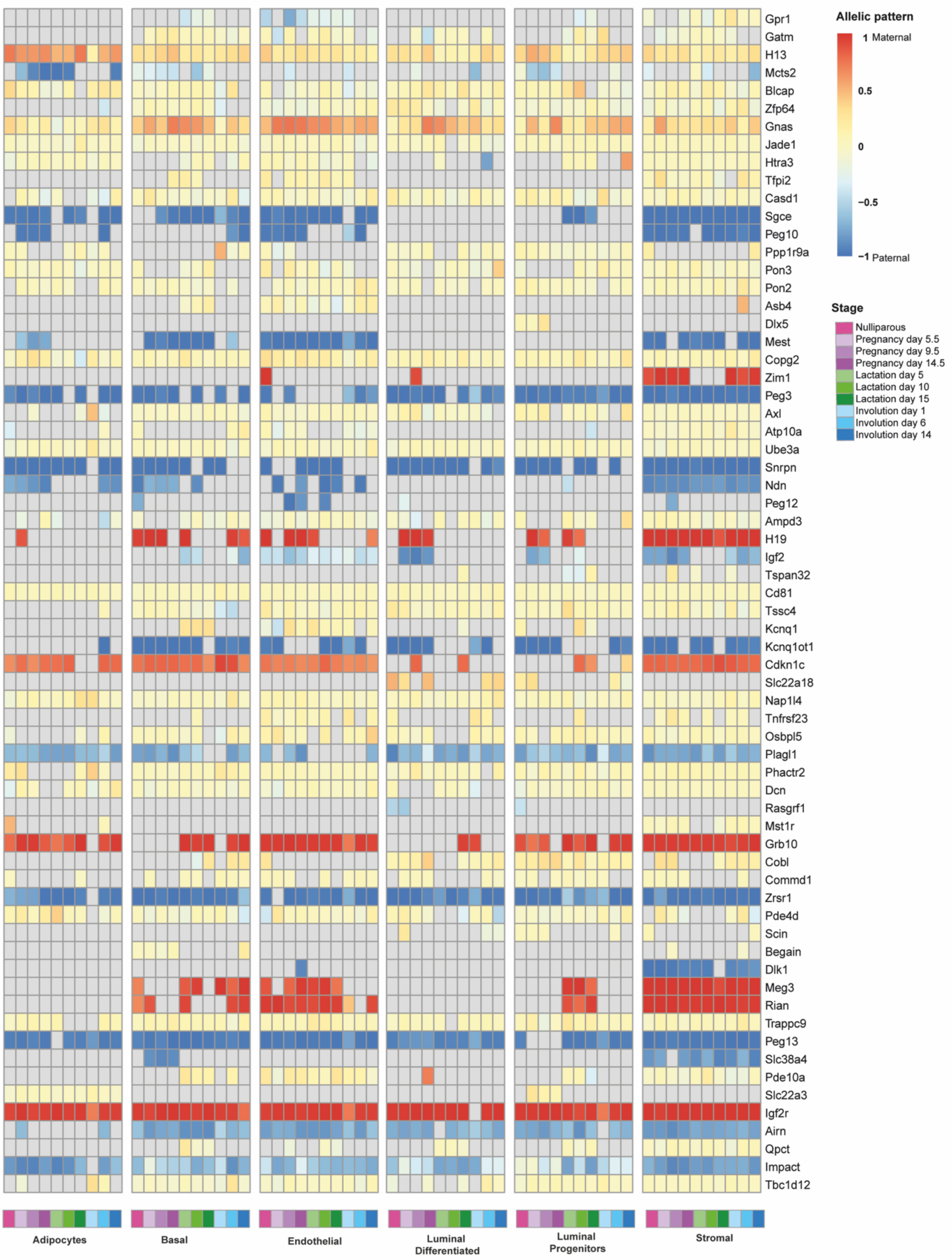

Heat map of allelic pattern arranged by chromosomal location, for all known imprinted genes which are expressed above a threshold of RPKM>1 in at least one cell type across the adult mammary gland developmental cycle. Maternal and paternal expression are

indicated in red and blue respectively, while yellow indicates biallelic expression. Grey indicated an expression level lower than the threshold. Each box represents an average of 8 biological replicates, 4 of each reciprocal cross.

Supplementary Figure 5 – Allelic expression patterns of placental, brain- and stage-specific, and weakly biased imprinted genes across mammary gland development

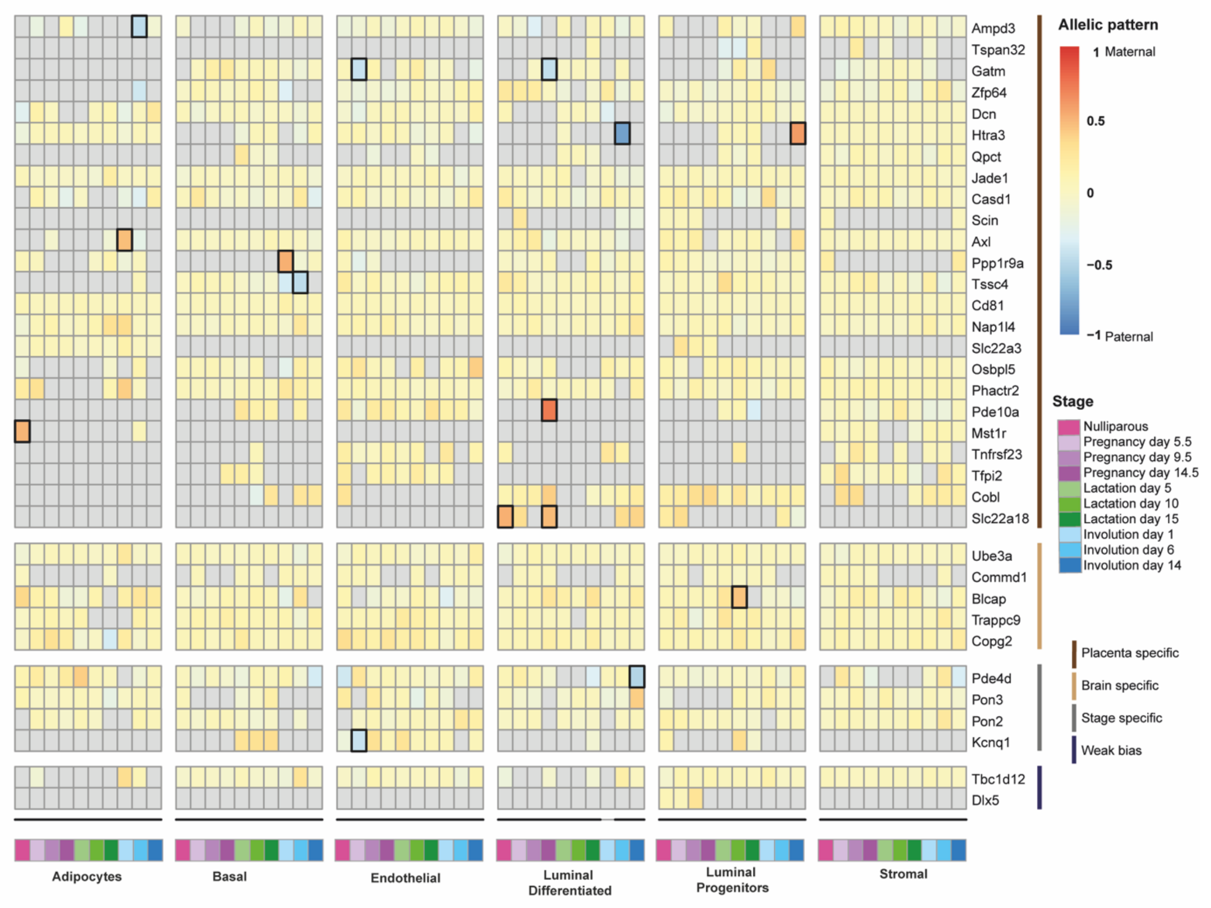

Heat map of allelic bias for imprinted genes known as placental, brain-specific, stage specific or displaying weak-bias, expressed above a threshold of RPKM>1 in at least one cell type across the adult mammary gland developmental cycle. Maternal and paternal expression are indicated in red and blue respectively, while yellow indicates biallelic expression. Grey indicated an expression level lower than the threshold. Boxed cells highlight values exceeding the 0.2 or 0.7 threshold for monoallelic expression, and each box represents an average of 8 biological replicates, 4 of each reciprocal cross.

Supplementary Figure 6 – Genome-wide allelic expression patterns across mammary gland development.

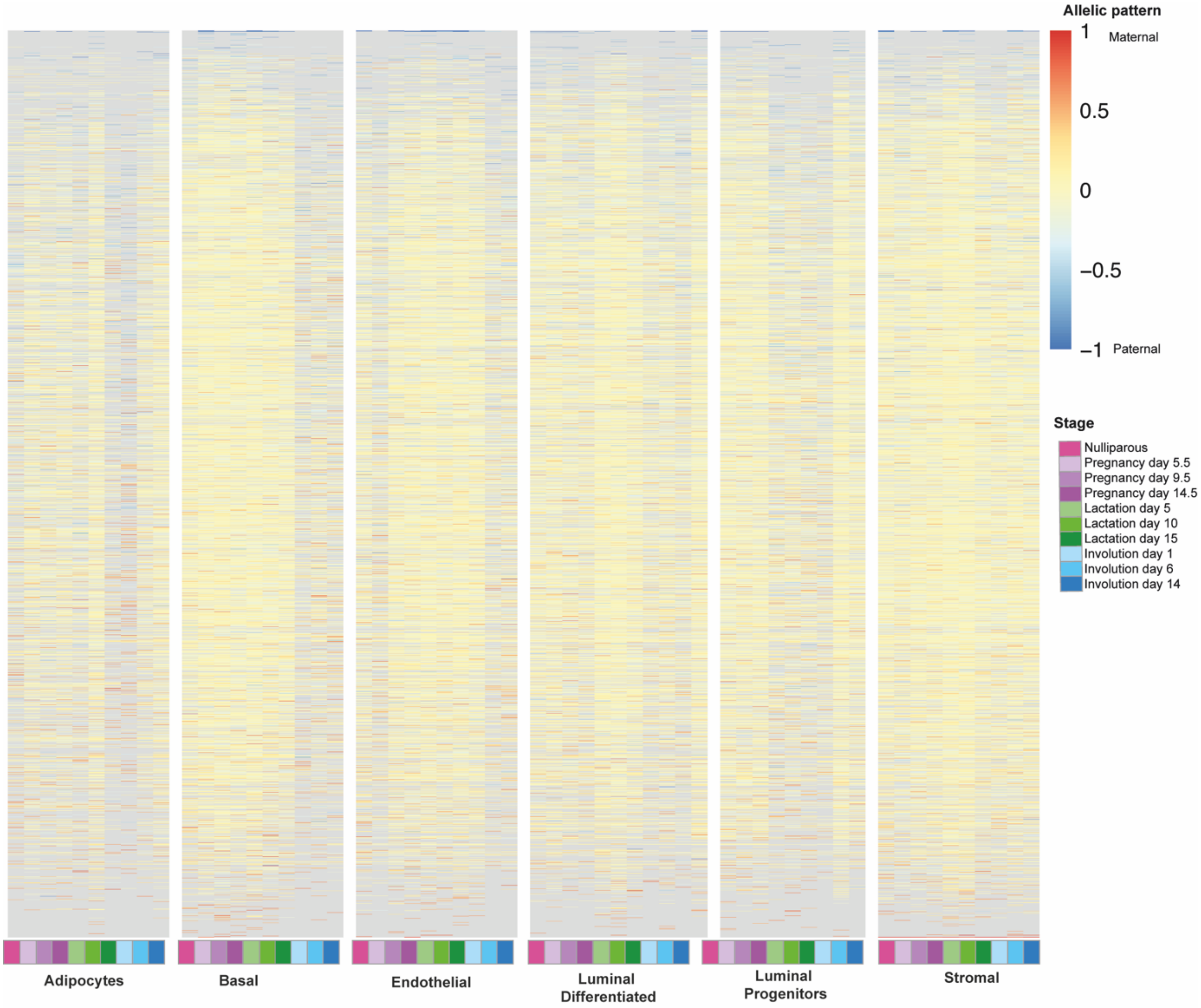

Heat map of allelic pattern of all genes expressed above a threshold of RPKM>1 in at least one cell type across the adult mammary gland developmental cycle, excluding all known imprinted genes Maternal and paternal expression are indicated in red and blue respectively, while yellow indicates biallelic expression. Grey indicated an expression level lower than the threshold. Each box represents an average of 8 biological replicates, 4 of each reciprocal cross.

Supplementary Figure 7 – Strain-specific allelic expression bias in the mammary gland

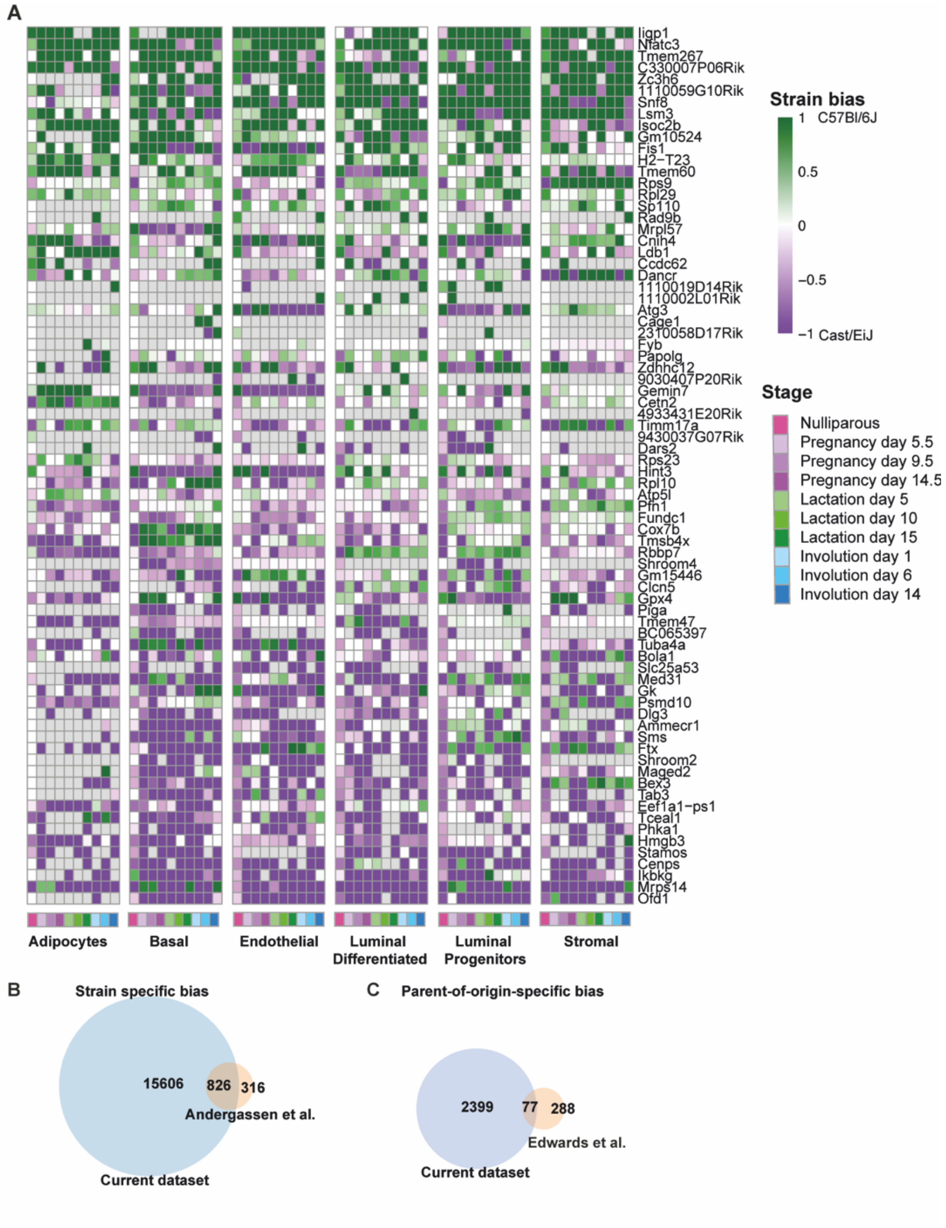

(A) Heat map showing statistically significant strain-specific bias between C57Bl6/J and CAST/EiJ with RPKM>1 in at least 3 timepoint or cell type as well as a bias greater than 0.5. **(B-C)** Venn diagram comparing the number of genes with strain-specific bias identified in this study compared to Andergassen et al. **(B)** or parent of origin-specific bias Edwards et al. 2023 **(C)**.
