# Supplementary material for: Dynamic allelic expression in mouse mammary glands across the adult developmental cycle
Source: Nucleic Acids Res. 2025 Sep 9;53(17):gkaf804. doi: 10.1093/nar/gkaf804 (PMC12419806; doi:10.1093/nar/gkaf804)
Supplement: gkaf804_Supplemental_Files [file gkaf804_supplemental_files.zip › Supplementary table 13 - primers.pdf]

Supplementary table 13 – Primers used in this study.
qPCR primers

Gene	Forward primer	Reverse Primer
PDGFRa	TCCTTCTACCACCTCAGCGAG	CCGGATGGTCACTCTTTAGGAAG
Krt5	TCTGCCATCACCCCATCTGT	CCTCCGCCAGAACTGTAGGA
Krt8	ACTCACTAGCCCTGGCTTCA	TCTTCACAACCACAGCCTTG
CD31	ACGCTGGTGCTCTATGCAAG	TCAGTTGCTGCCCATTCATCA
Adipoq	TGTTCCTCTTAATCCTGCCCA	CCAACCTGCACAAGTTCCCT
β-Tubulin	TTCAGCTGACCCACTCACTG	AGACAGGGTGGCATTGTAGG

Pyro

Gene	Forward primer	Reverse Primer	Sequencing Primer
Cdkn1c	TAGCAGGAACCGGAGATGG	[Btn] ACACCTTGGGACCAGCGTACT	TGGAAATCTGAAAACTGT
Meg3	CTCCTGGATTAGGCCAAAGC	[Btn] GGCCAGGGTCCAGAGTCTT	GACCCTCCAACTGTAAA
H19	GGGGGGTAGGATATATGTATTTTT	[Btn]ACCTCATAAAACCCATAACTATAAAATCAT	GTGTGTAAAGATTAGGG
Dlk1	[Btn]CGCAAGAAGAAGAACCTCCTGT	ACGCTGCTTAGATCTCCTCATCA	CAGCCTCCTTGTTGAA
Igf2	TCACGTCCCACACTAAGATCTCTC	[Btn]GGGGTGTCAATTGGGTTGT	AAGGGGATCTCAGCA
Snrpn	TAAATCTCAGCCCTTCTCTTCCC	[Btn]AATGCAGTAAGAGGGGTCAAAAA	CCCTTCTCTTCCCCTA
