# Supplementary material for: Dynamic allelic expression in mouse mammary glands across the adult developmental cycle
Source: Nucleic Acids Res. 2025 Sep 9;53(17):gkaf804. doi: 10.1093/nar/gkaf804 (PMC12419806; doi:10.1093/nar/gkaf804)
Supplement: gkaf804_Supplemental_Files [file gkaf804_supplemental_files.zip › Supplementary table 20 - primers_Revised.docx]

**Supplementary table 20 – Primers used in this study.**

qPCR primers

| **Gene** | **Forward primer** | **Reverse Primer** | **Amplicon size (bp)** |
| --- | --- | --- | --- |
| PDGFRa | TCCTTCTACCACCTCAGCGAG | CCGGATGGTCACTCTTTAGGAAG | 103 |
| Krt5 | TCTGCCATCACCCCATCTGT | CCTCCGCCAGAACTGTAGGA | 173 |
| Krt8 | ACTCACTAGCCCTGGCTTCA | TCTTCACAACCACAGCCTTG | 110 |
| CD31 | ACGCTGGTGCTCTATGCAAG | TCAGTTGCTGCCCATTCATCA | 109 |
| Adipoq | TGTTCCTCTTAATCCTGCCCA | CCAACCTGCACAAGTTCCCT | 104 |
| β-Tubulin | TTCAGCTGACCCACTCACTG | AGACAGGGTGGCATTGTAGG | 167 |

Pyrosequencing

| **Gene** | **Forward primer** | **Reverse Primer** | **Sequencing Primer** |
| --- | --- | --- | --- |
| Cdkn1c | TAGCAGGAACCGGAGATGG | [Btn] ACACCTTGGGACCAGCGTACT | TGGAAATCTGAAAACTGT |
| Meg3 | CTCCTGGATTAGGCCAAAGC | [Btn] GGCCAGGGTCCAGAGTCTT | GACCCTCCAACTGTAAA |
| H19 | GGGGGGTAGGATATATGTATTTTT | [Btn]ACCTCATAAAACCCATAACTATAAAATCAT | GTGTGTAAAGATTAGGG |
| Dlk1 | [Btn]CGCAAGAAGAAGAACCTCCTGT | ACGCTGCTTAGATCTCCTCATCA | CAGCCTCCTTGTTGAA |
| Igf2 | TCACGTCCCACACTAAGATCTCTC | [Btn]GGGGTGTCAATTGGGTTGT | AAGGGGATCTCAGCA |
| Snrpn | TAAATCTCAGCCCTTCTCTTCCC | [Btn]AATGCAGTAAGAGGGGTCAAAAA | CCCTTCTCTTCCCCTA |
